# Incorporating Cache Management Behavior into Seed Dispersal: The Effect of Pericarp Removal on Acorn Germination

**DOI:** 10.1371/journal.pone.0092544

**Published:** 2014-03-19

**Authors:** Xianfeng Yi, Mingming Zhang, Andrew W. Bartlow, Zhong Dong

**Affiliations:** 1 College of Life Sciences, Jiangxi Normal University, Nanchang, Jiangxi, China; 2 College of Agriculture, Henan University of Science and Technology, Luoyang, Henan, China; 3 Department of Biology, University of Utah, Salt Lake City, Utah, United States of America; University of Tasmania, Australia

## Abstract

Selecting seeds for long-term storage is a key factor for food hoarding animals. Siberian chipmunks (*Tamias sibiricus*) remove the pericarp and scatter hoard sound acorns of *Quercus mongolica* over those that are insect-infested to maximize returns from caches. We have no knowledge of whether these chipmunks remove the pericarp from acorns of other species of oaks and if this behavior benefits seedling establishment. In this study, we tested whether Siberian chipmunks engage in this behavior with acorns of three other Chinese oak species, *Q. variabilis*, *Q. aliena* and *Q. serrata var. brevipetiolata*, and how the dispersal and germination of these acorns are affected. Our results show that when chipmunks were provided with sound and infested acorns of *Quercus variabilis*, *Q. aliena* and *Q. serrata var. brevipetiolata*, the two types were equally harvested and dispersed. This preference suggests that Siberian chipmunks are incapable of distinguishing between sound and insect-infested acorns. However, Siberian chipmunks removed the pericarp from acorns of these three oak species prior to dispersing and caching them. Consequently, significantly more sound acorns were scatter hoarded and more infested acorns were immediately consumed. Additionally, indoor germination experiments showed that pericarp removal by chipmunks promoted acorn germination while artificial removal showed no significant effect. Our results show that pericarp removal allows Siberian chipmunks to effectively discriminate against insect-infested acorns and may represent an adaptive behavior for cache management. Because of the germination patterns of pericarp-removed acorns, we argue that the foraging behavior of Siberian chipmunks could have potential impacts on the dispersal and germination of acorns from various oak species.

## Introduction

Seed dispersal is an important ecological process that influences plant regeneration, community composition, and ecosystem diversity [1]–[4]. Trees bearing large fruits and seeds often depend on mammals and birds for dispersal of their seeds [5]–[6] to increase the probability of seedling recruitment far from parent trees [7]–[10]. Although a large proportion of seeds are eaten and killed by seed consuming animals, some are cached and may eventually escape predation and result in seedling establishment if the seeds are not recovered [1], [11]–[13].

Seed hoarders rely on cached food to guarantee their survival and reproduction during times of food shortage [14], [15]. However, the benefit of hoarding seeds will be reduced or even negated by invertebrates (e.g., weevils) that cause seed mortality [16]–[18]. Seed perishability by insects cause substantial losses to caches; therefore, a variety of behavioral adaptations of rodents exist for maximizing returns from cached seeds [16], [17], [19], [20]. Many studies show that seed hoarding animals prefer intact non-infested seeds over those infested by insects [20]–[26], reflecting their ability to distinguish between the two types. However, other studies show that rodents are incapable of discriminating against infested seeds [27]–[30].

Unlike most hoarding animals, Siberian chipmunks (*Tamias sibiricus*) select sound *Quercus mongolica* acorns by removing the pericarp and preferentially scatter hoarding them [31]. Most recently, we found that Pere David’s Rock squirrels (*Sciurotamias daviansis*) and Korean field mice (*Apodemus peninsulae*) remove pericarps before caching acorns of *Q. aliena* in central China (Yi’s personal observation). Previous studies have indicated that damage to acorns usually causes negative effects on acorn germination and seedling establishment [18], [31], [32]. However, pericarp removal and the release of the mechanical restriction of pericarps may promote acorn germination similar to the effects seen in sharp tooth oak (*Quercus aliena var. acuteserrata*) [33].

We have little knowledge of how pericarp removal by seed-hoarding animals influences acorn germination and seedling performance. Zhang [34] has reported that Siberian chipmunks remove the pericarps of *Q. liaotungensis* acorns in the Donglingshan mountains (Beijing) suggesting that the behavior is not restricted to *Q. mongolica*. Whether pericarp removal by Siberian chipmunks is performed on other oak species and represents a universal behavior, remains unknown. To understand how cache management strategy can influence the dispersal of oaks, we sought to determine the extent of this pericarp removal behavior using acorns of three white oak species (*Q. variabilis*, *Q. aliena,* and *Q. serrata var. brevipetiolata*) and to understand how this behavior affects acorn germination and seedling performance. We specifically aimed to test two hypotheses: 1) Siberian chipmunks will remove the pericarps from acorns of other white oak species and selectively cache those that are sound; and 2) pericarp removal by Siberian chipmunks will promote acorn germination and benefit seedling performance.

## Materials and Methods

### Ethics Statement

The Dailing Forestry Bureau of Heilongjiang Province issued permits for the experimental animal collection. To ensure the ethical treatment of animals throughout the study, our behavioral trials and housing procedures were approved by the College of Agriculture, Henan University of Science and Technology (No. 2011014).

### Study Site

The study was conducted in the Dongfanghong Forestry Center (average elevation 750 m, located at 46°50′∼46°59′N, 128°57′∼129°17′E) in Heilongjiang Province, northeastern China. The climate of the experimental site is dominated by the north temperate zonal monsoon with long, severe winters and short summers. The annual average temperature is 1.4°C with a maximum of 37°C and minimum of –40°C. The average annual precipitation is 660 mm, with 80% of the precipitation falling between May and September [31].

### Establishment of Enclosures

Separate semi-natural enclosures (10 m×10 m×2.5 m) were established in an open and level area. The enclosures were built using bricks and were 2.5 m above ground with a foundation 0.5 m into the ground. The walls of the enclosures were smoothed to prevent escape of small rodents. To prevent predators from entering, the tops of the enclosures were covered with plastic nets. An artificial nest constructed of bricks (H×W×L = 20 cm×15 cm×30 cm) and a plastic water bowl were placed at one corner of each enclosure to allow animals to rest and drink freely. A seed station measuring 0.5 m^2^ was established at the center of each enclosure. See Yi et al. [31] for more information about the semi-natural enclosures.

### Trapping of Animals

Steel frame live traps (H×W×L = 9 cm×10 cm×25 cm) baited with peanut seeds were placed on four transects at 5 m intervals in the field at 0900 hrs. Live traps were checked every three hours to ensure safety of the captured rodents. All traps were removed at 1600 hrs and returned the next morning. Trapping stopped on days with heavy rain. Captured Siberian chipmunks were transported by vehicle to laboratory housing within 30 minutes. Siberian chipmunks transported to the lab were kept individually in steel frame cages (H×W×L: 40 cm×50 cm×90 cm) with a natural temperature range (15–25°C) and photoperiod (14 hrs. of light). They were provided with carrots, peanuts, tree seeds, and water ad libitum. No animals perished during the trapping and laboratory feeding processes. All animals were released where they were captured following the experiments.

### Acorn Selection

To test whether Siberian chipmunks show a consistent response to insect-infested acorns of different oak species, acorns of *Q. variabilis*, *Q. aliena* and *Q. serrata var. brevipetiolata* were collected from more than 20 trees using seed traps. We used water flotation and visual inspection to determine whether acorns were infested [20]. Sound acorns had no apparent oviposition hole on the smooth pericarps, while infested acorns were identified by oviposition holes, irregular apophysis on the pericarps, and no sign of weevil larvae (*Curculio* spp.) emergence [30]. In total, we selected 200 sound and 200 infested acorns of each oak species (*Q. variabilis*, *Q. aliena*, and *Q. serrata var. brevipetiolata*) for experimental tests.

### Acorn Removal and Caching by Chipmunks

To test whether Siberian chipmunks rely on pericarp removal to distinguish between sound and infested acorns for scatter hoarding, eight *T. sibiricus* (4♀, 4♂; average body mass: 104.80±9.25 g) were randomly selected for acorn removal experiments in the semi-natural enclosures after one week of acclimation. One animal was introduced in each enclosure and provided with 20 sound and 20 infested larva-concealed acorns at 0700 hrs. Acorns were labeled with plastic tags according to Yi et al. [31]. Seed fates of the tagged acorns in the enclosures were determined in the afternoon of the same day (1700 hrs.). Sound and infested acorns of *Q. variabilis* were supplied to each individual first. The remaining acorns and debris were cleaned before the trials of *Q. aliena* and *Q. serrata var. brevipetiolata*. Acorns of *Q. variabilis*, *Q. aliena*, and *Q. serrata var. brevipetiolata* were all tested separately within a one-week period with 24 hrs between trials. Seed fates were defined as: intact in situ (IS), eaten in situ (EIS), eaten after dispersal (EAD), uneaten on the ground after dispersal (on surface) (UAD), and cached after dispersal (CAD).

### Germination of Pericarp-Removed Acorns

To test the effect of pericarp removal on acorn germination and seedling performance, 50 intact acorns of each oak species were randomly selected and used as a control group. For the treatment group, we were able to recover 50 pericarp-removed acorns of each oak species from caches of Siberian chipmunks. In November 2011, both intact and pericarp-removed acorns of each oak species were randomly sowed 1 cm deep into two plastic trays each with a 5×10 grid containing organic composite soil. Time to germination and germination rates for these acorns were measured. Dry masses of the epicotyls and roots of the seedlings were recorded 127 days after cultivation for each oak species.

To discriminate between the effects of pericarp removal and infestation by pre-dispersal seed predators on germination, we artificially shelled 25 randomly selected acorns of each oak species and planted them with 25 intact acorns in three plastic trays with a 5×10 grid. Plastic trays were kept in the laboratory at room temperature (20–25°C) and subject to visible light 800 μmol m^−2 ^s^−1^ radiation under a 14 h photoperiod. Plant containers were regularly watered to keep moist. An acorn was considered germinated when the epicotyl emerged, and time to germination was defined as the time when the first epicotyl emerged in each acorn group since planting. Germination success was recorded every few days after sowing.

### Data Analysis

Statistical Package for the Social Sciences (SPSS 16.0) and the statistical software R [35] was used for data analyses. Levene’s test was used to compare the variances among the variables. Linear models were used to detect seed fate differences between sound and infested acorns of the three oak species. The proportion germinated and time to germination of intact and pericarp-removed acorns were detected using Chi-square tests. The root and epicotyl biomasses of seedlings that originated from the intact acorns and those with the pericarp removed were tested for normality using the Kolmogorov-Smirnov test. Both were square root transformed to meet assumptions of normality for parametric tests. Differences in epicotyl and root biomasses between intact and pericarp removed seedlings were tested using two-sample t-tests. Data from this study were deposited in College of Agriculture, Henan University of Science and Technology and can be retrieved in the future.

## Results

**Figure 1:**
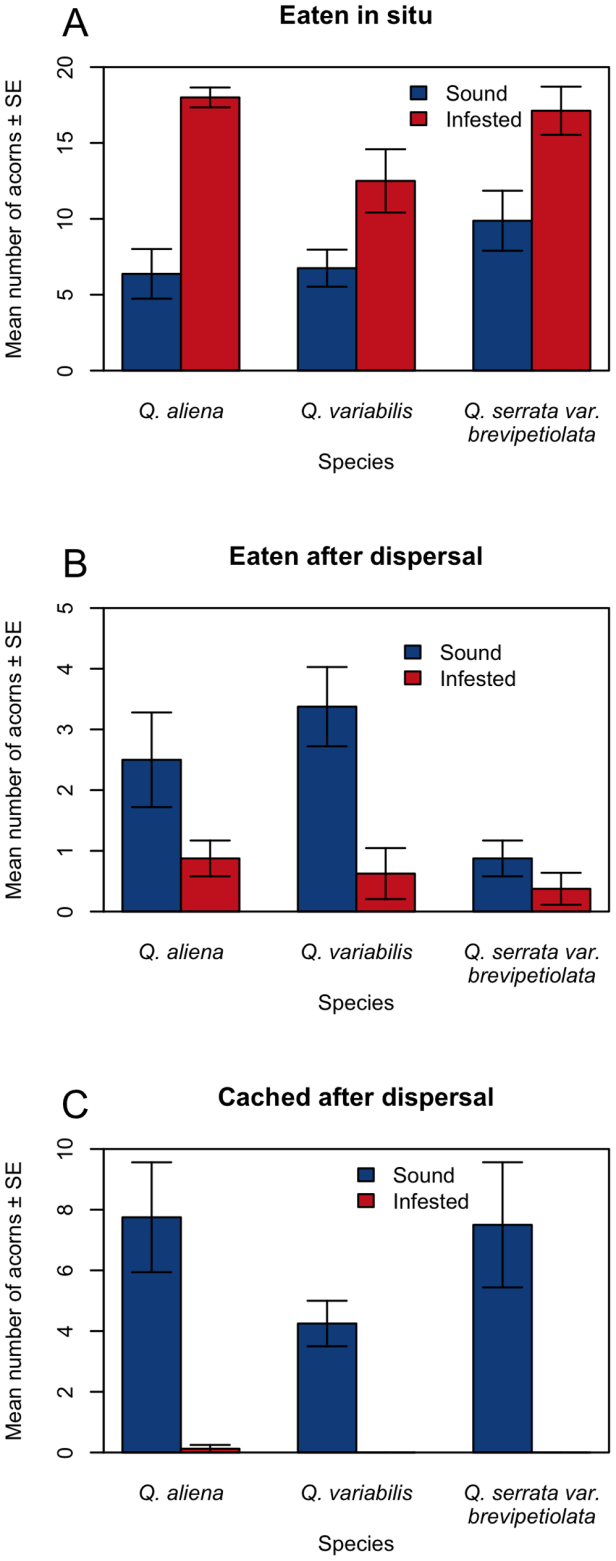
Seed fate of sound and infested acorns of *Q. variabilis*, *Q. aliena,* and *Q. serrata var. brevipetiolata* after being manipulated by Siberian chipmunks. A, B, and C indicate eaten in situ (EIS); eaten after dispersal (EAD), and cached after dispersal (CAD). Data are expressed as mean ± SE.

Sound and infested acorns of *Q. variabilis*, *Q. aliena*, and *Q. serrata var. brevipetiolata* were removed at equal rates at seed stations (*Q. variabilis*: F = 0.145, df = 1, P = 0.709; *Q. aliena:* F = 2.411, df = 1, P = 0.143; *Q. serrata var. brevipetiolata*: F = 0.338, df = 1, P = 0.570) (Figure 1A, B, C). These results suggest that Siberian chipmunks do not preferentially select sound acorns from the seed stations when presented with both infested and sound seeds. Siberian chipmunks removed the entire pericarps of all acorns before they dispersed and/or cached them after removal from seed stations. Consequently, more sound acorns were removed and scatter hoarded (CAD) than infested acorns of all three oak species (*Q. variabilis*: F = 32.111, df = 1, P<0.001; *Q. aliena*: F = 17.659, df = 1, P = 0.001; *Q. serrata var. brevipetiolata*: F = 13.24, df = 1, P = 0.003) (Figure 1). The infested acorns were more likely to be eaten at seed stations (EIS) than the sound acorns (*Q. variabilis*: F = 5.653, df = 1, P = 0.032; *Q. aliena*: F = 43.525, df = 1, P<0.001; *Q. serrata var. brevipetiolata*: F = 8.182, df = 1, P = 0.013) (Figure 1). In addition, more sound acorns of *Q. variabilis* were found to be eaten after dispersal (EAD) than the infested acorns (F = 12.548, df = 1, P = 0.003) (Figure 1), but those results were not found in acorns of *Q. aliena* and *Q. serrata var. brevipetiolata* (*Q. aliena*: F = 3.804, df = 1, P = 0.071; *Q. serrata var. brevipetiolata*: F = 1.600, df = 1, P = 0.227) (Figure 1). Infestation did not affect the number of acorns remaining intact after dispersal (UAD) (all P>0.05). When pooling the numbers of EAD, UAD and CAD acorns together, we found that the sound acorns were more likely to be dispersed out of the seed stations than the infested acorns (*Q. variabilis*: F = 31.003, df = 1, P<0.001; *Q. aliena*: F = 16.409, df = 1, P<0.001; *Q. serrata var. brevipetiolata*: F = 13.389, df = 1, P = 0.003).

**Figure 2:**
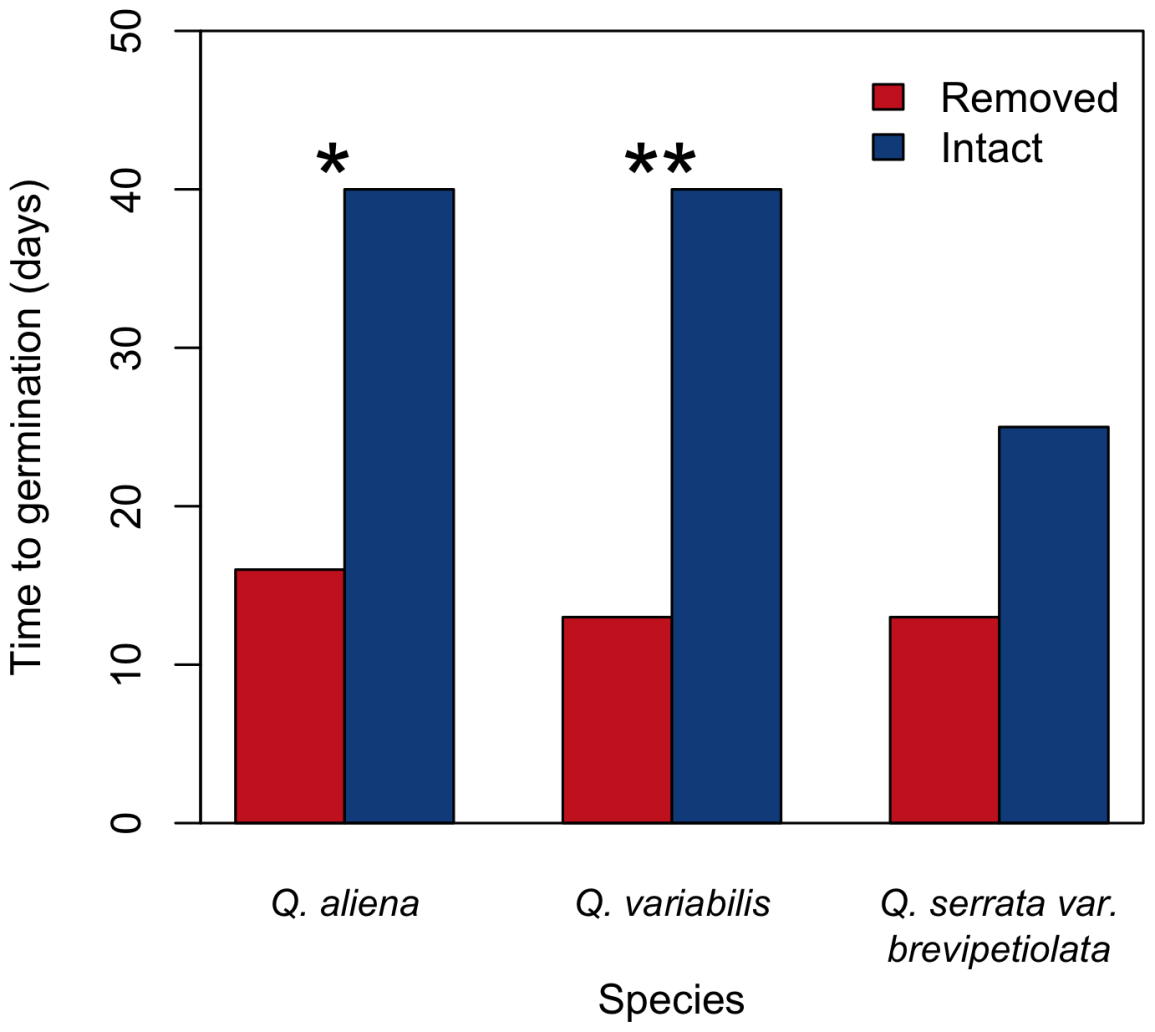
Time to germination of three oak species germinated from intact acorns and those with pericarps removed by Siberian chipmunks. Time to germination was the time at which the first acorn in each group germinated. Chi-squared tests were performed for each species, and asterisks indicate significance (**P<0.001, *<0.01).

**Figure 3:**
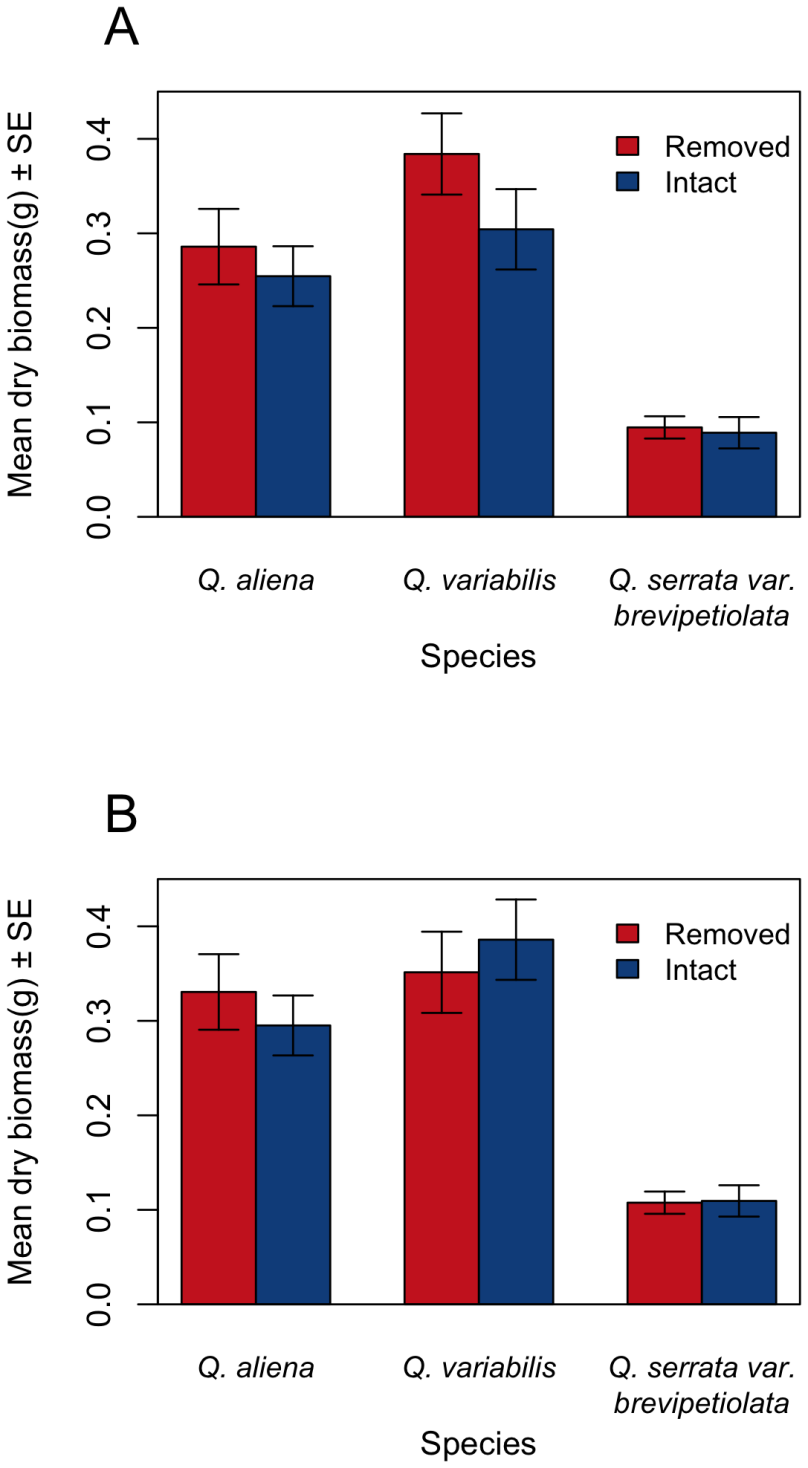
Dry masses of the epicotyls (A) and roots (B) of seedlings of three oak species germinated from intact acorns and those with pericarps removed by Siberian chipmunks. All the dry masses of the epicotyls and roots were not significant between intact and pericarp removed acorns for all species (P>0.05).

The acorn germination results indicate that time to germination of pericarp-removed acorns was shorter than that of intact acorns for *Q. variabilis*, *Q. aliena*, and *Q. serrata var. brevipetiolata* (*χ^2^* = 13.755, df = 1, *P*<0.001; *χ^2^* = 10.286, df = 1, *P* = 0.001; *χ^2^* = 3.789, df = 1, *P* = 0.052, respectively) (Figure 2). Acorns with the pericarp removed by Siberian chipmunks exhibited higher germination rates than intact acorns for *Q. variabilis* (*χ^2^* = 6.002, df = 1, *P* = 0.014), *Q. aliena* (*χ^2^* = 9.849, df = 1, *P* = 0.0017), and *Q. serrata var. brevipetiolata* (*χ^2^* = 10.704, df = 1, *P* = 0.001) (Table 1). However, no significant difference was found in the germination rates between the intact acorns and those that pericarps were removed artificially, except for *Q. aliena* (*Q. variabilis*: *χ^2^* = 1.039, df = 1, *P* = 0.308, *Q. aliena*: *χ^2^* = 4.720, df = 1, *P* = 0.0298; *Q. serrata var. brevipetiolata*: *χ^2^* = 0.04, df = 1, *P* = 0.842; Table 1). No significant difference was found in the epicotyl biomass of seedlings between the intact acorns and those with pericarp removed by Siberian chipmunks (*Q. variabilis*: t = 1.273, df = 35.852, *P* = 0.211; *Q. aliena*: t = 0.778, df = 45.967, *P = *0.441; *Q. serrata var. brevipetiolata*: t = 0.265, df = 23.724, *P* = 0.794; Figure 3A). We also failed to see significant differences in the root dry masses of seedlings originated from the intact acorns and those with pericarp removed by Siberian chipmunks (*Q. variabilis*: t = −0.609, df = 35.361, *P* = 0.546; *Q. aliena*: t = 0.572, df = 44.578, *P* = 0.570; *Q. serrata var. brevipetiolata*: t = −0.186, df = 20.593, *P* = 0.854; Figure 3B).

**Table 1:** The percentage of acorns that germinated in each species when pericarps were removed and when acorns were intact.

	Field study		Experimental study	
Species	Pericarp removed (n = 50)	Acorn intact (n = 50)	Pericarp removed (n = 25)	Acorn intact (n = 25)
*Q. aliena*	92^a^	64^b^	88^a^	68^b^
*Q. variabilis*	84^a^	60^b^	76^a^	86^a^
*Q. serrata var. brevipetiolata*	72^a^	36^b^	48^a^	52^a^

Acorns in the field study had the pericarps removed by Siberian chipmunks, while the acorns in the experimental study had the pericarps artificially removed. Different letters in the same row indicate significance (P<0.05) for the field and experimental studies.

## Discussion

Acorns of oaks are often infested by various invertebrate insects (e.g., weevils and moths), resulting in the loss of acorn production and failure of oak regeneration [36]–[39]. Weevil larvae in acorns will continuously consume tissue making acorns much more vulnerable to infection by fungi and bacteria [40], [41]. Our results show that Siberian chipmunks remove the pericarp from acorns of three more white oak species and selectively consume infested acorns and cache those that are sound. It appears that Siberian chipmunks do not determine whether or not an acorn is sound until after removing the pericarp. They then determine the appropriate action for that particular seed (eat, cache, etc.). These findings provide further support that Siberian chipmunks remove the pericarps of these three species to discriminate against infested seeds similar to *Q. mongolica* [31].

Siberian chipmunks in the present study are different from larder hoarding eastern chipmunks (*Tamias striatus*), which usually remove the pericarps of chestnut oak (*Q. montana*) acorns to consume the weevil larvae inside (Yi’s personal observation). Despite these behavioral differences, selective consumption of weevil-infested acorns not only allows Siberian chipmunks to overcome cache losses [31], [42], [43], it may also provide a large amount of protein to counter high tannins in acorns [27], [44]–[47]. Therefore, selectively caching sound acorns may help Siberian chipmunks gain maximum rewards from their caches [17], [20], [31].

Our study shows that pericarp-removed acorns germinate more rapidly than intact ones consistent with previous studies on *Quercus* spp. that show that partial damage to acorns can promote germination [33], [38], [48]–[50]. The early germination of pericarp-removed acorns may be explained by mechanical or physiological responses to seed damage, such as increased water intake by cotyledons or a decrease in potential germination inhibitors in the pericarps [33]. In addition, our results demonstrate that pericarp removal by Siberian chipmunks increases acorn germination of the three oak species. The pericarp-removed acorns had significantly higher germination than those of intact acorns. This may be due to a proportion of the intact acorns being infested by insect feeders that dramatically decrease germination [37]–[39]. Furthermore, the pericarp-removed acorns we selected were all sound (see Methods) because Siberian chipmunks selectively cache sound acorns after removing the pericarps [31]. This is supported by the fact that the germination of intact and artificially shelled acorns were not significantly different from one another. Nevertheless, we cannot exclude the effects of pericarps on acorn germination [33]. We admit that field conditions largely differ from laboratory conditions, especially in biological interactions. Removing pericarps may increase the risk of infection by *Ciboria* spp., an acorn specific decomposing fungi, reported to cause serious mortality to acorns [51], [52]. However, a previous study shows that pericarp removal exhibits no negative effect on acorn germination of *Q. mongolica* in natural conditions [31]. This implies that removing pericarps does not cause serious fungal infection possibly because of high tannin levels or other defense compounds in acorns [32].

Pericarp removal shows no significant effects on seedling performance in terms of dry masses of epicotyls and roots of seedlings, slightly different from the results of Liu et al. [33]. This may be because of mechanical constraints rather than chemical inhibitors. It is generally accepted that epicotyl dormancy of acorns in the field is mainly caused by inhibitors in cotyledons, embryos, or pericarps [33], [53]. However, intact acorns of *Q. variabilis* and *Q. aliena* in this study exhibited high germination rates (64% and 60%, respectively) at high cultivation temperatures (20–25°C), implying that epicotyl dormancy is more likely to be temperature-dependent rather than physiologically inhibited.

We have shown that sound acorns are preferentially cached by Siberian chipmunks and that pericarp removal can influence germination of acorns and seedling performance. Rapid acorn germination of white oaks has been regarded as an adaptation to escape predation by rodents [54]. We predict that pericarp-removed acorns are more likely to escape predation by food hoarding animals than intact acorns due to faster germination. Additionally, pericarp removal by Siberian chipmunks may be beneficial because sound acorns show a greater ability to germinate than those that are infested [36], [37], [39]. Moreover, Siberian chipmunks remove the pericarps and selectively consume infested acorns, enhancing acorn dispersal and simultaneously suppressing insect populations infesting oaks. Even though acorn consumption results in the loss of viable propagules, long term consequences of pericarp removal by Siberian chipmunks, and possibly other food hoarding animals (e.g., squirrels and mice), may benefit seed dispersal, seed germination, and seedling establishment of several oak species.
