# Correction: Incorporating Cache Management Behavior into Seed Dispersal: The Effect of Pericarp Removal on Acorn Germination

**DOI:** 10.1371/journal.pone.0104726

**Published:** 2014-08-01

**Authors:** The PLOS ONE Staff

There are errors in Table 1 and the legend of Figure 3. The correct statistics for the experimental study shown in Table 1 are as follows:

*Q. variabilis*: *χ^2^*  =  0.125, df  =  1, *P*  =  0.724; *Q. aliena*: *χ^2^*  =  1.865, df  =  1, *P*  =  0.172; *Q. serrata var. brevipetiolata*: *χ^2^*  =  0, df  =  1, *P*  =  1.

The authors have provided the corrected versions of Table 1 and Figure Legend 3, which can be viewed here.

**Figure 3:**
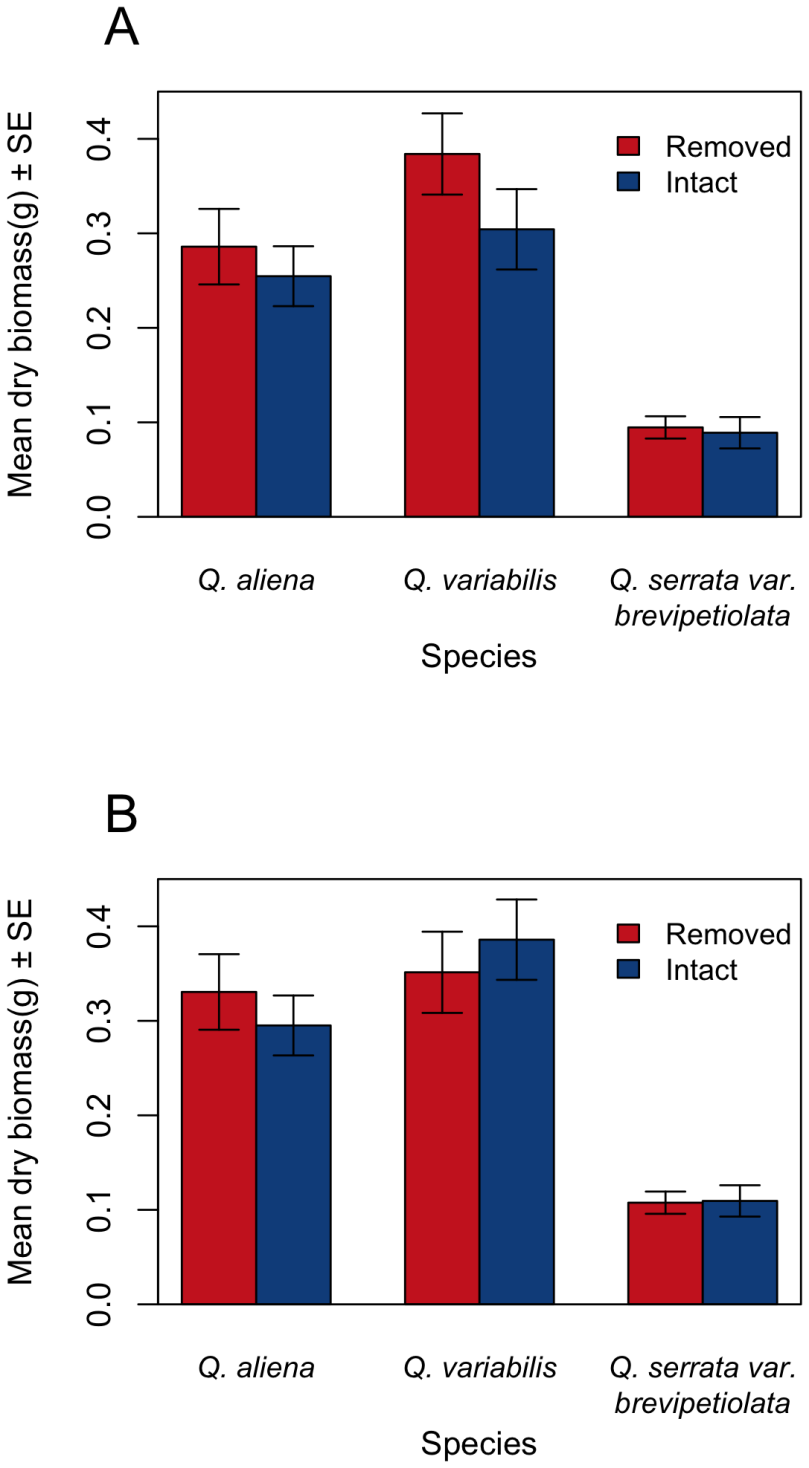
Dry masses of the roots (A) and epicotyls (B) of seedlings of three oak species germinated from intact acorns and those with pericarps removed by Siberian chipmunks. All the dry masses of the epicotyls and roots were not significant between intact and pericarp-removed acorns for all species (P > 0.05).

**Table 1:** The percentage of acorns that germinated in each species when pericarps were removed and when acorns were intact.

	Field study		Experimental study	
Species	Pericarp removed (n = 50)	Acorn intact (n = 50)	Pericarp removed (n = 25)	Acorn intact (n = 25)
*Q. aliena*	92^a^	64^b^	88^a^	68^a^
*Q. variabilis*	84^a^	60^b^	76^a^	84^a^
*Q. serrata var. brevipetiolata*	72^a^	36^b^	48^a^	52^a^

Acorns in the field study had the pericarps removed by Siberian chipmunks, while the acorns in the experimental study had the pericarps artificially removed. Different letters in the same row indicate significance (P<0.05) for the field and experimental studies.
